# Tryphactothripini of India (Thysanoptera, Thripidae, Panchaetothripinae), with identification keys and a new record of *Opimothrips*

**DOI:** 10.3897/zookeys.884.39500

**Published:** 2019-10-30

**Authors:** Remani R. Rachana, Laurence A. Mound, Shashikant G. Rayar

**Affiliations:** 1 Department of Entomology, University of Agricultural Sciences, Dharwad, Karnataka 580001, India; 2 Division of Germplasm Collection and Characterization, National Bureau of Agricultural Insect Resources (ICAR-NBAIR), Bengaluru, Karnataka 560024, India; 3 Australian National Insect Collection, CSIRO, Canberra, Australia

**Keywords:** *
Astrothrips
*, *Opimothrips tubulatus*

## Abstract

An identification key is provided to the four genera of Panchaetothripinae from India that are members of the Tribe Tryphactothripini, together with a key to identify all the known six Indian species of *Astrothrips*. Furthermore, the genus *Opimothrips* is newly reported from India.

## Introduction

The Thripidae subfamily Panchaetothripinae currently comprises approximately 145 species in 42 genera (ThripsWiki 2019). These species breed on the leaves of a wide range of plants in tropical countries, and include the widespread pest, the Greenhouse Thrips, *Heliothrips haemorrhoidalis* (Bouché) (Xie et al. 2019). Within this subfamily is a tribe, Tryphactothripini, that comprises species in which the second abdominal tergite is sharply constricted anteriorly and bears complex sculpture anterolaterally. This sculpture is in the form of closely spaced, small ridges that often give the optical impression of being stout, sharply recurved, microtrichia. Twenty species in nine genera are currently placed in this tribe, and four of these genera, with nine species, are recorded from India.

Members of this tribe are restricted to the tropics, with a few *Astrothrips* species also found in subtropical areas. Despite being leaf feeders, a few adults have also been taken from grass. Recently, an additional genus of Tryphactothripini was found in India, *Opimothrips*. This monotypic genus was described from Thailand and also recently reported from China (Nonaka and Okajima 1992; Xie et al. 2019). The objective here is to give an account of the tribe Tryphactothripini in India and to report a new record of genus *Opimothrips* with a note on *O. tubulatus*.

## Taxonomy

### Key to genera of Tryphactothripini from India

**Table:** 

1	Fore wing distal half with costal setae longer than fringe cilia	*** Noathrips ***
–	Fore wing distal half with costal setae shorter than fringe cilia	**2**
2	Genae without protruding fringe of pale sculpture; fore wing uniformly shaded with apex sharply paler	*** Opimothrips ***
–	Genae with protruding fringe of pale sculpture, fore wing with dark and light bands	**3**
3	Abdominal tergites III–VII with paired clusters of round sculptured areolae, striated across anterior third, bearing sigmoidal setae	Tryphactothrips ^*^
–	Abdominal tergites III–VII without paired clusters of round sculptured areolae; reticulated across anterior third, sigmoidal setae present or absent	*** Astrothrips ***

#### Astrothrips

Taxon classificationAnimaliaThysanopteraThripidae

Karny, 1921

B3DD1FEE-DB02-5495-862F-18DBA31CF8D1

Astrothrips Karny, 1921: 239. Type species Heliothrips globiceps Karny, 1913.

##### Notes.

This genus was based originally on a single species that had been described from a single female collected in New Guinea, but four generic synonyms are listed in ThripsWiki (2019).

It is an Old World genus that is restricted to tropical countries, with two species from Africa and 10 distributed between Pakistan and New Guinea (ThripsWiki 2019). These are leaf-feeding thrips, with the occasional adult found in flowers, and although adults have been taken from a wide variety of plants, suggesting the possibility of polyphagy, larvae remain unknown for most of the described species. The many published host-plant associations (Table 1) involve more than 30 plant families, with little indication of any specificity. These records are based on the collection of one or more adults from any given plant, and thus may involve a flying adult simply resting on a plant surface without feeding. Possibly these records are more of a measure of the dispersive behavior of adults, rather than an indication of the plants on which they might breed. Moreover, most of the species are known from few specimens. This combination of small sample size and lack of biological information leads to a lack of confidence in the relatively trivial structural differences that have been used to distinguish some of the species. The species of *Astrothrips* from India were reviewed by Bhatti (1967), with many clear illustrations in the form of line drawings, and Ananthakrishnan and Sen (1980) provided a further key to the species from India but without illustrations.

**Table 1.** *Astrothrips* distributions and host-associations of adults. Data from: Akram (2000); Kudô (1979, 1995); Mirab-balou et al. 2011; Reyes (1994); Saeed and Yousuf (1994); Tillekaratne et al. (2011).

Species	No. of antennal segments	Distribution	Host associations	Larvae found
*A. asiaticus*	8	India	*Colocasia*; *Lantana*; *Ricinus*;*Wedelia*; “weeds”	No
*A. aucubae*	7	China; Japan; Philippines	*Aucuba*; *Thalictrum*	No
*A. aureolus*	5	Malaysia; Australia	* Hymenocallis *	No
*A. bhattii*	5	Nigeria	*Citrus*; *Colocasia*; *Cucurbita*; “palm”	On *Citrus grandis*
*A. chisinliaoensis*	6	Malaysia; China; Taiwan	*Canthium*; *Carallia*; *Morus*; *Rhus*	No
*A. globiceps*	6	India; Myanmar; Malaya; Thailand; Philippines; New Britain; New Guinea; Japan	*Angiopteris*; *Calopogonium*; *Centrosema*; *Colocasia*; *Crinum*; *Dysoxylum*; *Hyptis*; *Lantana*; *Microdesmis*; *Pleocnemia*; *Plumeria*; *Portulaca*; “bamboo”	No
*A. lantana*	7	India; Nepal	*Lantana*; *Quercus*; “evergreen tree”	No
*A. parvilimbus*	6	India	*Antirrhinum*; *Boerhavia*; *Crinum*; *Erythrina*; *Ipomoea*; *Musa*; *Ricinus*; *Sida*; “fern”	No
*A. roboris*	5	Nigeria; Ghana; Sudan	*Colocasia*; *Cucurbita*; *Musa*; *Nicotiana*; *Phaseolus*; *Piper*; *Thunbergia*; “climbing legume” “palm”	On *Colocasia esculenta*
*A. stannardi*	7	India; Pakistan	*Celosia*; *Ipomoea*; *Lantana*; *Mirabilis*; *Verbascum*	No
*A. strasseni*	8	Myanmar; China	“bamboo”	No
*A. tumiceps*	7	India, Pakistan; Sri Lanka; Malaysia; Myanmar; Indonesia; Philippines; Thailand; Japan; Australia	*Artocarpus*; *Canna*; *Centrosema*; *Dysoxylum*; *Dolichos*; *Eleusine*; *Erythrina*; *Glycine*; *Gossypium*; *Lantana*; *Mallotus*; *Phaseolus*; *Ricinus*; “fern”	No

##### Diagnosis.

Small, dark brown, strongly reticulate Panchaetothripinae usually with banded fore wings bearing stout veinal setae. Antennae with 5–8 segments, sense cones on III and IV forked or simple. Head with conspicuous raised reticulate sculpture, setae all small, ocellar area sometimes elevated; maxillary palps bi-segmented. Pronotum transverse, strongly sculptured with some raised reticulation. Mesoscutum usually deeply notched; metascutum with prominent triangle. Tarsi 1-segmented. Fore wing slender, both longitudinal veins with prominent setae; costal setae shorter than costal cilia; posteromarginal cilia wavy. Tergite II of abdomen with anterior margin strongly constricted, and anterolaterally with a group of prominent strongly recurved microtrichia; tergites III–VII with transverse reticulation on anterior half; VIII with no posteromarginal comb; X divided longitudinally. Male smaller, sternites with or without slender, deeply curved, pore plates.

##### Antennal segmentation.

The 8-segmented condition of antennae is considered plesiotypic for the family Thripidae (Zhang et al. 2019). However, among some Panchaetothripinae genera, including *Astrothrips*, there are species with the distal segments fused in different combinations, such as segments VI+VII or VII+VIII, or sometimes VI–VIII or even V–VIII forming a terminal group. As a result of this fusion the number of apparent segments is reduced to seven, six, or even five. It is important to recognise that reduction in the number of segments is not necessarily a shared apomorphy, because the 7-segmented condition could arise by fusion of either VII+VIII or by VI+VII (Zhang et al. 2019).

##### Species recognition.

Species level taxonomy in this genus is based on some relatively trivial character states, each of which may have been observed on very few specimens. Stannard and Mitri (1962) described *aureolus* from only two females and distinguished this new species from *globiceps* and *parvilimbus*. The three diagnostic characters selected by the authors (shape of antennal segment III, colour of costal setae, body colour) are now considered to be variable among more recently collected specimens. Some other character states used by authors to distinguish species in this genus have been found to be more variable with the discovery of more specimens. Bhatti (1967) described *stannardi* as having the major sense cone on antennal segment VI surpassing the apex of the antenna, but this has been found to be untrue on various specimens of the species collected from South India and Thailand. Wilson (1975) in his key to species treated *stannardi* in the group with five or six antennal segments, but in the main text under that species, he states that there are seven segments; this confusion is repeated by Ananthakrishnan and Sen (1980). Subsequent identifications that are based solely on such original descriptions may not be reliable. The male of *aureolus* has been unknown, but a male identified as this species from Timor Leste (in ANIC) has U-shaped pore plates on sternites IV–VII as in *stannardi*. Similar problems are involved in host-plant associations. For example, Bhatti (1967) described *lantana* from two females, but Wilson (1975) mentions weekly collections from *Lantana camara* near the type locality without finding this thrips. In contrast, Kudô (1995) identified three females from Nepal as *lantana* that were taken from a species of *Quercus* and an unidentified tree.

### Key to Indian species of *Astrothrips*

**Table:** 

1	Antennae 8 segmented; segments separated by clear sutures	***A. asiaticus***
–	Antennae with 5 to 7 segments	**2**
2	Pronotum posterior margin without a sub-marginal transverse apodeme [male with no sternal pore plates]	***A. tumiceps***
–	Pronotum posterior margin with distinct transverse sub-marginal apodeme	**3**
3	Pronotal sub-marginal transverse apodeme weak, present only on median third (Fig. 7); posterolateral angles of pronotum with raised sculpture	***A. globiceps***
–	Pronotal sub-marginal transverse apodeme strong, extending fully across pronotum (Fig. 8); no raised sculpture on pronotal posterolateral angles	**4**
4	Antennae 6-segmented; mesoscutal anterior notch shorter than median intact part [male with pore plates on sternites V–VII]	***A. parvilimbus***
–	Antennae 7-segmented	**5**
5	Antennal segments V–VII separated by clear sutures; male not known	A. lantana^*^
–	Antennal segments V–VII not separated by clear sutures; male with pore plates on sternites IV–VII	***A. stannardi***

#### Noathrips

Taxon classificationAnimaliaThysanopteraThripidae

Bhatti, 1967

385A7401-545A-5836-A281-93E6353183D2

Noathrips Bhatti, 1967: 9. Type species Noathrips prakashi Bhatti, 1967 by monotypy.

##### Notes.

The genus was erected with the type species *prakashi*, collected on herbage and *Lantana* leaves from Jabalpur, Madhya Pradesh (Bhatti 1967). Later, this species was collected in Sri Lanka and China (Kudô 1979; Xie et al. 2019).

##### Diagnosis.

Antennae 8-segmented, III and IV with forked sense cones. Head without conspicuous raised structure, postocular seta 4 strongly developed, ocellar hump small; maxillary palps bi-segmented. Pronotum with weakly raised sculpture. Mesoscutum not notched anteriorly; metascutum with prominent reticulate triangle. Tarsi 1-segmented. Fore wing with slender pointed setae; costal setae longer than fringe cilia; posteromarginal setae wavy. Abdominal tergite I with a postmarginal flange; II anterior margin constricted, with narrow plate like cuticular processes laterally; III–VII with transverse reticulations on anterior half, posterior half smooth; X asymmetrical, divided longitudinally. Males smaller; sternites IV–VII each with transversely elongated anteriorly concave pore plates.

#### Opimothrips

Taxon classificationAnimaliaThysanopteraThripidae

Nonaka & Okajima, 1992

3E487122-85CB-578E-A5A3-E6D7FFFF923C

Opimothrips Nonaka and Okajima 1992: 106. Type species: Opimothrips tubulatus Nonaka & Okajima, 1992 by monotypy.

##### Notes.

The reports of *O. tubulatus* from Thailand and China are from grass, and hence Xie et al. (2019) reported the species as being associated with grasses. However, the present specimens have been collected from an unidentified weed.

##### Diagnosis.

Antennae 8-segmented, III and IV with thin, Y-shaped sense cones with the arms curving around the segment. Head polygonally reticulate, cheeks constricted at base; maxillary palps bi-segmented. Pronotum uniformly reticulate, two pairs of campaniform sensilla, one pair of long setae. Mesoscutum entire; metascutum with reticulate triangle. Tarsi 1-segmented. Fore wing with prominent veins, costal setae shorter than fringe cilia; posteromarginal setae wavy. Abdominal tergite I reticulate, median pair of setae minute; II strongly constricted, wart-like tubercles laterally; III–VII with thick antecostal line; X asymmetrical, divided longitudinally.

#### Opimothrips tubulatus

Taxon classificationAnimaliaThysanopteraThripidae

Nonaka & Okajima, 1992

FD399F42-4A62-5C63-847D-CEC6490E803D

Figures 1–6

##### Material studied.

Three females, Chitradurga, Karnataka, India, on unidentified weed, 04 December 2017, Rachana R.R. leg. Two females deposited in the Insect Museum, National Bureau of Agricultural Insect Resources (ICAR-NBAIR), Bengaluru, India. One female deposited in ANIC – Australian National Insect Collection, CSIRO, Canberra.

##### Female macroptera.

**Figures 1–6.**
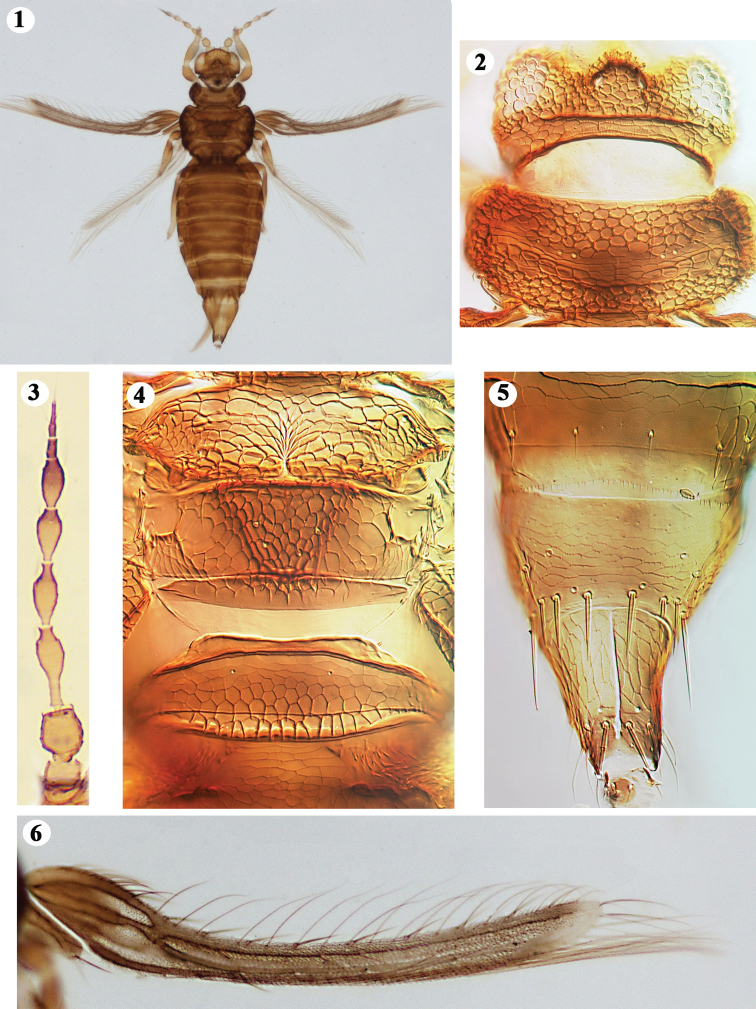
*Opimothrips tubulatus***1** female **2** head and prothorax **3** antenna **4** pterothorax and female abdominal tergite I **5** female abdominal tergites IX–X **6** fore wing.

Body yellowish brown (Fig. 1), fore legs yellow, tarsi yellow, tibiae brown, yellow in apical half and basally, femora brown in basal half, rest yellow; antennal segments I–III golden yellow, IV–V yellow with shaded brown distally, VI–VIII dark brown; fore wing uniformly shaded with apex pale, clavus brown; first vein with 11 setae, not uniformly arranged; second vein with 6 setae. Antennae 8-segmented; sutures complete and distinct between all segments; III–IV with thin, Y-shaped sense cones, the arms unusually thin and curving around the segment, narrow apex on IV shorter, wider, more abruptly constricted than III; outer sense cone on VI extending to midpoint of VIII (Fig. 3). Head wider than long; ocellar hump weakly developed, ocelli visible; major setae rudimentary; eyes not bulged, covering lateral side almost completely; genae much reduced, without protruding transparent fringe (Fig. 2). Pronotum reticulate, raised sculpture on lateral margins; median area with transverse reticulations (Fig. 2). Mesonotum anterior margin shallowly notched, not reaching beyond anterior one third; 2 pairs of small setae, the inner pair anterior to the outer pair (Fig. 4). Metanotal median triangle weakly indicated; polygonally reticulate, extending beyond posterior margin, median setae anterior to campaniform sensilla (Fig. 4). Fore wing base humped, costal setae shorter than fringe; first vein with 7 basal setae, 2 at middle and 2 distally, thin and pointed; second vein with 6 setae, curved except last three; clavus with 4 veinal setae but no discal seta; posteromarginal cilia wavy (Fig. 6). Fore tibia with a spine at apex; hind tibia with a row of 11 conspicuous spines on inner side and two stout ones at apex; hind tarsi with a spine at median on inner side and two short, stout ones at apex. Abdominal tergite I reticulations extending beyond margin; median area of II with weak reticulations, laterally with wart-like tubercles; thick sublateral antecostal line on III–VII, laterally forming a posterior directed notch; VIII with complete posteromarginal comb of minute teeth; IX with campaniform sensilla; X asymmetric, median split complete, terminal setae almost half as long as the segment (Fig. 5). Sternites II–VII with 2 pairs of marginal setae on broad craspedum; antecostal lines on III–VII with median concave invagination. Ovipositor long, well developed, exceeding abdominal apex.

#### Tryphactothrips

Taxon classificationAnimaliaThysanopteraThripidae

Bagnall

CD57598A-91B2-5496-82E1-3CE61E42F278

Tryphactothrips Bagnall, 1919: 256. Type species Dinurothrips rutherfordi Bagnall, 1915, by original designation.

##### Notes.

Various workers on Thysanoptera [Bagnall (1919); Ramakrishna and Margabandhu (1931, 1940); Shumsher (1947); Patel and Patel (1953); Ananthakrishnan (1954); Wilson (1975)] reported *T. rutherfordi* from India. However, Bhatti (1967 and 1990) clarified the report of Ramakrishna and Margabandhu (1931) as *Astrothrips tumiceps* and of Patel and Patel (1953) from Pune as a species of *Astrothrips*. The records by Shumsher (1947) and Ananthakrishnan (1954) have never been validated, but Wilson (1975) collected three females from a forest tree in Tamil Nadu and compared these with the female holotype of *T. rutherfordi* in London. This is the only authenticated report of *rutherfordi* from India, and is only the second reliable report since the original description of the species from Ceylon.

##### Diagnosis.

Antennae with six segments, terminal segments fused into an elongate unit. Head with raised sculpture covering cheeks and vertex; maxillary palps bi-segmented. Pronotum with raised sculpture anteriorly and on anterolateral angles. Mesoscutum slightly notched. Tarsi 1-segmented. Fore wing with anterior vein fused to costa at fork of veins; costal setae shorter than costal cilia; posteromarginal cilia wavy. Abdominal tergite II sharply constricted, laterally with double based rod like processes; III–VII with paired clusters of round sculptured areolae, striated across anterior third, bearing pair of sigmoidal setae laterally; VIII with no posteromarginal comb; X asymmetric, divided longitudinally.

**Figures 7, 8.**
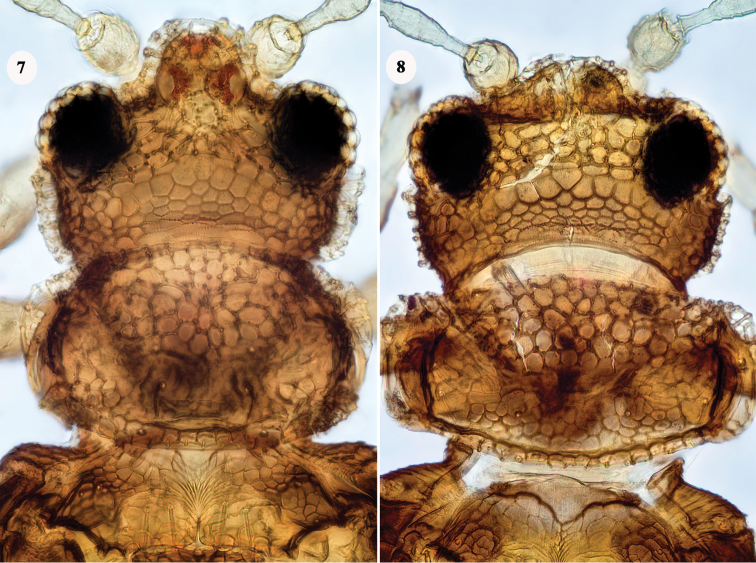
*Astrothrips* species, head and pronotum **7***globiceps*; female from Java, compared to holotype and identified by J.S. Bhatti **8***parvilimbus*; female from Madras on *Erythrina*, identified by T.N. Ananthakrishnan. [images by Manfred Ulitzka].

## Supplementary Material

XML Treatment for Astrothrips

XML Treatment for Noathrips

XML Treatment for Opimothrips

XML Treatment for Opimothrips tubulatus

XML Treatment for Tryphactothrips

## Footnotes

* From original description

* from original description
